# BEST: Improved Prediction of B-Cell Epitopes from Antigen Sequences

**DOI:** 10.1371/journal.pone.0040104

**Published:** 2012-06-27

**Authors:** Jianzhao Gao, Eshel Faraggi, Yaoqi Zhou, Jishou Ruan, Lukasz Kurgan

**Affiliations:** 1 School of Mathematical Sciences and LPMC, Nankai University, Tianjin, People's Republic of China; 2 School of Informatics, Indiana University Purdue University, Indianapolis, Indiana, United States of America; 3 Center for Computational Biology and Bioinformatics, Indiana University School of Medicine, Indianapolis, Indiana, United States of America; 4 State Key Laboratory of Medicinal Chemical Biology, Nankai University, Tianjin, People's Republic of China; 5 Department of Electrical and Computer Engineering, University of Alberta, Edmonton, Alberta, Canada; University of South Florida College of Medicine, United States of America

## Abstract

Accurate identification of immunogenic regions in a given antigen chain is a difficult and actively pursued problem. Although accurate predictors for T-cell epitopes are already in place, the prediction of the B-cell epitopes requires further research. We overview the available approaches for the prediction of B-cell epitopes and propose a novel and accurate sequence-based solution. Our BEST (B-cell Epitope prediction using Support vector machine Tool) method predicts epitopes from antigen sequences, in contrast to some method that predict only from short sequence fragments, using a new architecture based on averaging selected scores generated from sliding 20-mers by a Support Vector Machine (SVM). The SVM predictor utilizes a comprehensive and custom designed set of inputs generated by combining information derived from the chain, sequence conservation, similarity to known (training) epitopes, and predicted secondary structure and relative solvent accessibility. Empirical evaluation on benchmark datasets demonstrates that BEST outperforms several modern sequence-based B-cell epitope predictors including ABCPred, method by Chen et al. (2007), BCPred, COBEpro, BayesB, and CBTOPE, when considering the predictions from antigen chains and from the chain fragments. Our method obtains a cross-validated area under the receiver operating characteristic curve (AUC) for the fragment-based prediction at 0.81 and 0.85, depending on the dataset. The AUCs of BEST on the benchmark sets of full antigen chains equal 0.57 and 0.6, which is significantly and slightly better than the next best method we tested. We also present case studies to contrast the propensity profiles generated by BEST and several other methods.

## Introduction

Identification of immunogenic regions/segments in a given antigen protein chain finds important applications in immunotherapies [1], [2]. Experimental search for these regions is work and resource intensive and would benefit from guidance offered by computational methods that accurately identify these segments. Although such accurate methods are already in place for the prediction of T-cell epitopes [3], [4], further research is needed to develop accurate predictors of the B-cell epitopes [3], [5]. The B-cell epitopes are categorized into continuous (linear) and discontinuous (conformational). The majority of B-cell epitopes are conformational [6], however, the computational approaches concentrate mostly on the prediction of “easier” linear epitopes [3], [7].

The first attempts to predict the antigenic determinants concerning linear B-cell epitopes from protein chains date back to the 1980s [8]–[12]. These methods were relatively simple, monoparametric (based on a single propensity such as hydrophilicity), and were limited to small protein datasets. In the 1990s, researchers investigated the usefulness of multiple propensities including hydrophilicity, solvent accessibility, flexibility, and secondary structure propensities, for the B-cell epitope prediction [6], [13]–[15]. Results generated in these works were used to develop the BEPITOPE method [16], which combines multiple propensities. The predictive quality of single propensity-based methods was critically evaluated by Blythe and Flower [5], which motivated further development in this area. The last decade observed an influx of new methods that use more advanced models for the prediction of the linear epitopes. The BepiPred method [17] applies a hidden Markov model which takes two propensity scores as its inputs. A number of machine learning-based model were recently developed, from decision trees and k-nearest neighbor that utilized a combination of multiple propensities and sequence complexity as inputs [18], to neural network-based ABCPred [19] that performs predictions directly from protein chain. The later method is designed to recognize epitopic peptides with 20 or fewer (i.e., 10,12,14,16 and 20) amino acids (AAs). The newest sequence-based predictors of continuous B-cell epitopes exclusively use support vector machine (SVM) models. They include: (1) a method by Chen et al. [20] that predicts 20-mer peptides using a new AA pair-based antigenicity scale [20]; (2) BCPred [21] that predict the 12, 14, 16, 18, 20, and 22-mer long epitopes directly from sequence using a new type of string kernel-based SVM; (3) COBEpro [22] which utilizes a two-stage design with an SVM that takes novel sequence similarity scores as inputs to predict variable-size peptides in the first stage and a second stage that combines these fragments to predict epitopes in full chains; and (4) BayesB method [23] that predicts epitopes of diverse lengths (from 12 to 20-mers) using position specific scoring matrix (PSSM) generated with PSI-BLAST [24]. We note that COBEpro was extended to predict conformational epitopes via its second stage. Moreover, one sequence-based method, CBTOPE [25], was proposed for the prediction of conformational epitopes. This is also an SVM-based predictor that utilizes multiple propensities and sequence-derived inputs including composition and collocation of AAs.

**Figure 1:**
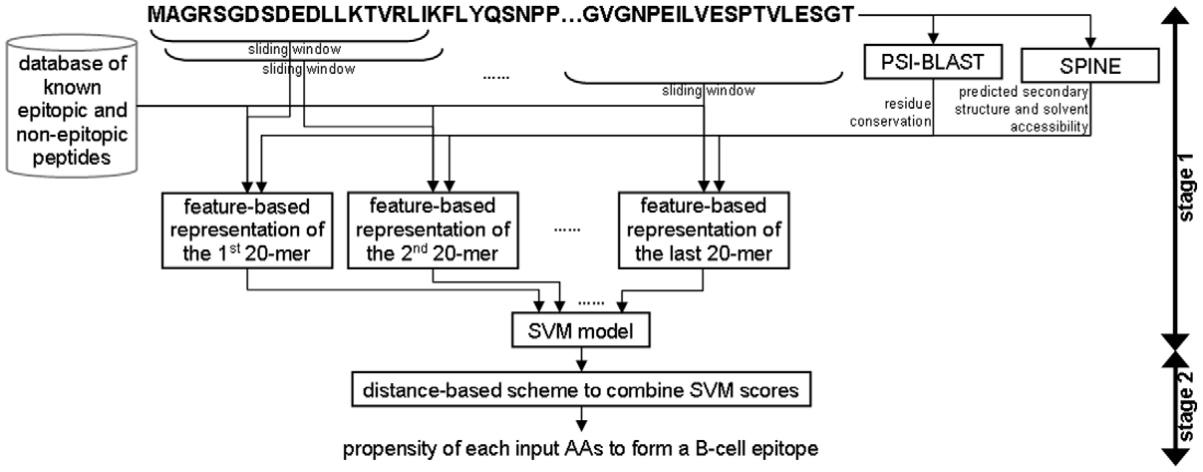
Overall design of the proposed BEST method.

There are also a few predictors that use protein structure as their input and which predict the conformational epitopes. Early structure-based methods use relatively simple scoring-based approaches. They include CEP [26] that is based on scoring surface AAs using their solvent accessibility, DiscoTope [27], which uses surface/solvent accessibility, contact numbers, and AA propensity scores, and SEPPA [28] that combines a new propensity score with information about solvent accessibility and the packing density of AAs. More recent methods use machine learning models to perform predictions. These include PEPITO [29] that applies linear regression to AA propensity scores and solvent accessibility quantified using half sphere exposure; EPSVR [30] that uses Support Vector Regression and several inputs including epitope propensity scores, contact numbers, secondary structure composition, conservation, side chain energy surface and planarity scores; a method by Zhang et al. [31], which utilizes random forest model; and a predictor by Liu and Hu [32] that uses logistic regression model and information concerning B-factors and relative accessible surface area. Moreover, in recent years two new types of approaches were developed. The first, called Bepar [33] is based on association patterns between antibody and antigen residues and the other, EPMeta [30], is a consensus-based method, which combines multiple discontinuous epitope predictors. Finally, Epitopia [34], [35] is a machine learning-based approach which utilizes Naïve Bayes to process information extracted based on physico-chemical and structural-geometrical properties from a surface patch defined using solvent accessibility. Since this method allows performing predictions from sequence alone, we include it in our comparative analysis.

**Table 1:** Summary of the considered features and features selected and used in the proposed sequence-based predictor of B-cell epitopes.

Feature group	Abbreviated name	Number of features	Number of selected features
Predicted secondary structure (SS)	SS	8	2
Predicted RSA	RA	33	5
RAAP score	RP	30	24
Conservation score	CS	29	2
Predicted SS and RSA	SS+RA	12	6
Predicted SS and conservation score	SS+CS	6	1
Predicted SS and RAAP score	SS+RP	6	1
RAAP score and predicted RSA	RP+RA	30	17
RAAP and conservation scores	RP+CS	28	18
Predicted SS and RSA, and RAAP score	SS+RA+RP	6	1
Similarity score	SIM	10	7
Total number of features		198	84

**Table 2:** Comparison of predictive quality on the BCPREDFrag dataset calculated using 10-fold cross validation. The methods are sorted by their AUC values in the ascending order.

Method	AUC	Accuracy	Sensitivity	Specificity	Precision	F-measure	MCC
Chen et al. [20] a	0.700	0.641	0.529	0.752	0.681	0.596	0.29
BCPred^a^	0.758	0.679	0.726	0.632	0.664	0.694	0.36
COBEpro^b^	0.768	0.714	0.554	0.874	0.815	0.660	0.45
SVM model 198^c^	0.811	0.745	0.561	0.929	0.887	0.687	0.53
SVM model 84^d^	0.813	0.740	0.495	0.984	0.969	0.655	0.55

a results from Table 1 in [21].

b results from Table II in [22].

c results for the SVM model (*C* = 8.0 and *gamma* = 0.000977) that uses all 198 features.

d results for the SVM model (*C* = 1.0 and *gamma* = 0.001953) that uses the selected 84 features.

Our aim is to develop an accurate computational model for the prediction of both linear and conformational epitopes based on an approach similar to COBEpro [22]. We design a novel two-stage scheme that predicts conformational and linear epitopes from antigen chains based on accurate predictions of linear epitopes from the first stage. The motivation for our design comes from the fact that current methods use a wide variety of diverse inputs. We hypothesize that improvements can be attained by combining these inputs. The novelty of our BEST (Bcell Epitope prediction using Support vector machine Tool) method is two-fold. First, we effectively use multiple inputs including sequence conservation calculated using outputs from PSI-BLAST, predicted solvent accessibility and secondary structure (SS), and certain propensity and sequence similarity scores. Some of these inputs are motivated by existing works [20], [22], [23], [34], [35]. However, we are the first to propose a sequence-based method that uses the residue conservation scores (conservation was previously used to build the structure-based EPSVR predictor [30]) and to generate novel descriptors/features that combine multiple inputs, such as SS and conservation, SS and an antigenicity scale, solvent accessibility and conservation, etc. Second, we use a novel design of the second stage that utilizes a sliding window based on predictions of linear epitopes to compute propensities for formation of epitopes (both linear and conformational) for all residues in the input antigen sequence. This allows for more practical applications, in contrast to some other solutions, such as ABCPred [19], method by Chen et al. [20], BCPred [21], and BayesB [23], which predict only short peptide fragments. Moreover, we empirically demonstrate that BEST outperforms recent sequence-based solutions including the method by Chen et al. [20], BCPred [21], ABCPred [19], CBTOPE [25], and COBEpro [22].

**Table 3:** Comparison of predictive quality on the ChenFrag dataset calculated using either 10-fold cross validation or 5-fold cross validation to match the test type from the corresponding manuscripts. The methods are sorted by their AUC values in the ascending order.

Method	AUC	Accuracy	Sensitivity	Specificity	Precision	F-measure	MCC
Chen et al. [20] a	unavailable	0.725	0.636	0.765	0.701	0.667	0.40
SVM model 198^b^	0.835	0.783	0.587	0.979	0.966	0.730	0.62
COBEpro^c^	0.829	0.780	0.609	0.951	0.925	0.734	0.59
SVM model 198^d^	0.840	0.792	0.597	0.987	0.979	0.742	0.63
SVM model 84^e^	0.848	0.788	0.579	0.998	0.996	0.732	0.63

The methods are sorted by their AUC values in the ascending order.

a results based on 5-fold cross validation from Table 3 in [20].

b results based on 5-fold cross validation for the SVM model (*C* = 8.0 and *gamma* = 0.000977) that uses all 198 features.

c results based on 10-fold cross validation from Table I in [22].

d results based on 10-fold cross validation for the SVM model (*C* = 8.0 and *gamma* = 0.000977) that uses all 198 features.

e results based on 10-fold cross validation for the SVM model (*C* = 1.0 and *gamma* = 0.001953) that uses the selected 84 features.

## Methods

### Overview of the proposed B-cell epitope predictor

BEST utilizes a two-stage design, see Figure 1. In the first stage, we use a sliding window to represent the input antigen chain as a set of 20-mers. These 20-mers are encoded by a numerical feature vector that quantifies information in the window, which includes features extracted from

- The chain including AA propensity scale that was introduced in [20] and sequence similarity scores proposed in [22] against a database of known (training) epitopic and non-epitopic peptides.
- The evolutionary profile generated by PSI-BLAST including conservation scores calculated from the Weighted Observation Percentage (WOP) matrix.
- The secondary structure and solvent accessibility that are predicted from the input chain with SPINE [36], [37].

Motivated by the designs of recent predictors [20]–[23], [25], we apply an SVM-based model to predict epitopes using these features. In the second stage, we combine predictions from the SVM using a novel, custom-designed scheme that outputs the propensity of each AA to form of a B-cell epitope.

**Figure 2:**
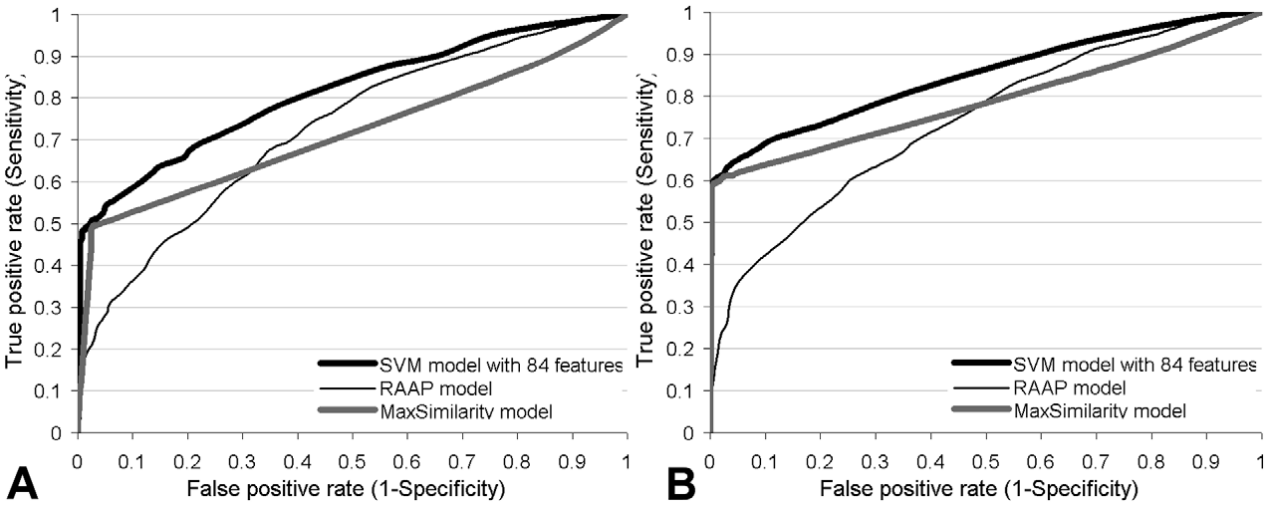
Receiver operating characteristic (ROC) curves for the SVM model with 84 features, RAAP and MaxSimilarity models. The curves were computed based on the 10-fold cross validation on the BCPREDFrag dataset (panel A) and ChenFrag dataset (panel B).

### Datasets and test protocols

We use two datasets composed of 20-mers. The ChenFrag dataset, which was introduced in [20], consists of 872 20-mers that are B-cell epitopes and 872 non-B-cell epitope 20-mers. The epitope 20-mers were generated by a truncation-and-extension from BciPep database [38] and the non-epitope fragments were taken from SWISS-PROT. The BCPREDFrag dataset was introduced in [21] and includes 701 epitopes 20-mers and 701 non-epitopes 20-mers. Originally, this dataset included 947 unique epitopes extracted from the BciPep database. After truncation-and-extension to 20-mers this set was no longer non-redundant. Therefore, they were processed using CD-HIT [39] to obtain a reduced set of 701 epitopes, which share at most 80% similarity. The non-epitopes were selected from SWISS-PROT. We use this dataset to design (select relevant features and parameterize the SVM) our predictive model using 10-fold cross validation. The final design (using the same parameters and features) is tested on the ChenFrag dataset using 10-fold cross validation. The use of the 10-fold cross validation is motivated by the fact that the same test protocol was used in prior works [21], [22].

**Table 4:** AUC values on the BCPREDFrag and ChenFrag datasets calculated using 10-fold cross validation obtained by using selected features from individual feature groups; abbreviates names of feature groups are given in Table 1.

Dataset	SS	RA	RP	CS	SS+RA	SS+CS	SS+RP	RP+RA	RP+CS	SS+RA+RP	SIM
BCPREDFrag	0.557	0.542	0.716	0.501	0.602	0.568	0.532	0.695	0.710	0.556	0.760
ChenFrag	0.565	0.547	0.743	0.496	0.584	0.545	0.555	0.738	0.743	0.560	0.824

We use an independent test set that was utilized as a test dataset in [34]. This dataset, which we call SEQ194, includes 194 protein sequences. Since the SEQ194 dataset was also derived from the BciPep database, we reduce the identity between SEQ194 and the BCPREDFrag dataset (which is used as our training/design dataset) to 40%. To do that, we remove any 20-mer from our training dataset that shares above 40% identity with any chain in SEQ194, and we call the resulting dataset Filtered40_BCPREDFrag. This dataset includes 633 20-mer fragments with 86 epitopic fragments and 547 non-epitopic fragments. When testing our method on the SEQ194, we build our predictor using the Filtered40_BCPREDFrag. This includes the use of the filtered version of the training dataset as a database of known epitopic and non-epitopic peptides for which we calculate the sequence similarity scores according to the method from [22].

**Figure 3:**
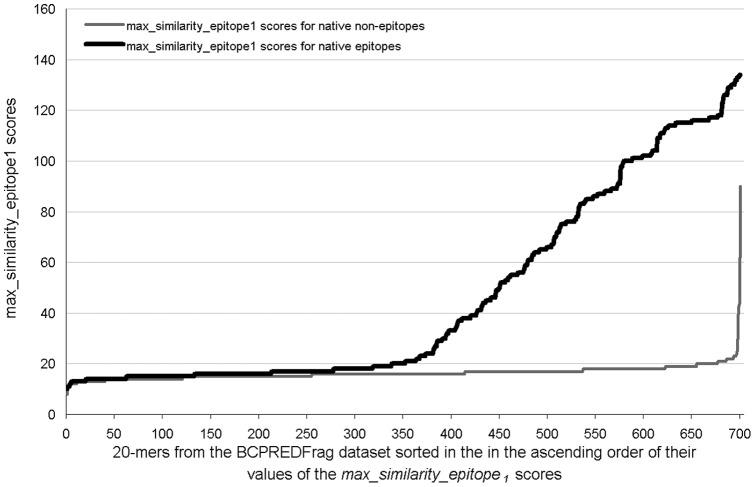
The values of the similarity-based scores between the 20-mers from the BCPREDFrag dataset and the library of the epitope fragments, i.e., the *max_similarity_epitope_1_* feature. The black line shows the similarity scores for the native epitope and the gray line for the non-epitope fragments. The *x*-axis corresponds to the sorted list (in the ascending order based on the similarity scores) of the 701 epitopic and 701 non-epitopic 20-mers from the BCPREDFrag dataset, and the *y*-axis shows their corresponding similarity scores.

We also use a second sequence-based test dataset called SEQ19, which includes 19 proteins and which was introduced in [30]. The dataset was extracted using Conformational Epitope Database [40] by considering entries with unbound antigen structures, no complex structures, and where multiple entries with the same antigen structure were combined (antigenic residues from multiple entries were mapped onto one structure). The pairwise sequence identity in this dataset was reduced to up to 35%.

The datasets are available at http://biomine.ece.ualberta.ca/BEST/.

**Figure 4:**
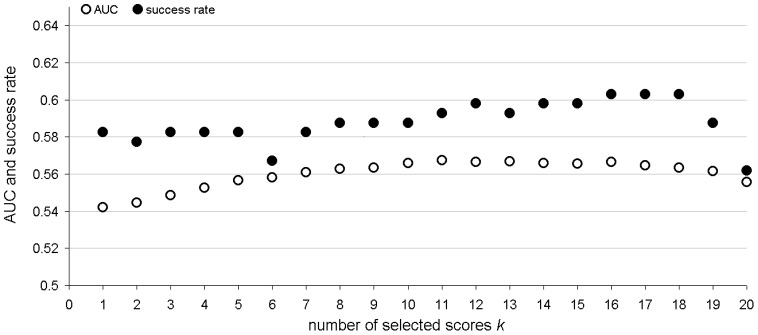
The AUC and success rate values in the function of the number of selected scores *k* (*x*-axis) when using SVM model with 84 features and the *distance scheme* to predict B-cell epitopes on the SEQ194 dataset. We use the Filtered40_BCPREDFrag to generate the SVM model.

**Table 5:** The AUC and success rate for the prediction of the B-cell epitopes on the SEQ194 dataset when using predictions from the SVM model with 84 features and the five schemes: *maximum, average, median, and distance scheme* with *k* = 10 and *k* = 16. We use the Filtered40_BCPREDFrag to generate the SVM model.

Method	Success rate	AUC
Max scheme	47.4%	0.52
Average scheme	56.2%	0.56
Median scheme	60.8%	0.55
Distance scheme *k* = 10	58.8%	0.57
Distance scheme *k* = 16	60.3%	0.57

### Evaluation of predictive quality

The predicted propensity of a given AA in the input protein chain is a real number which is (often) binarized to denote two outcomes: whether or not the residue is a part of an epitope. The evaluation of the binary predictions uses several quality measures including accuracy (ACC), sensitivity, specificity, precision, F-measure, and Matthews correlation coefficient (MCC):

Accuracy  =  (TP+TN)/(TP+FP+TN+FN)

Sensitivity  =  TP/(TP+FN)

Specificity  =  TN/(TN+FP)

Precision  =  TP/(TP+FP)

F-measure  = 2*TP/(2*TP+FN+FP)

MCC  =  (TP*TN+FP*FN)/sqrt{(TP+FP)*(TP+FN)*(TN+FP)*(TN+FN)}

where TP and TN are the number of correctly predicted epitope and non-epitope residues, respectively, FP is the number of non-epitope residues that were predicted to be in an epitope, and FN is the number of epitope residues that were predicted not to be in an epitope. Higher values of these measures indicate better quality of predictions.

**Figure 5:**
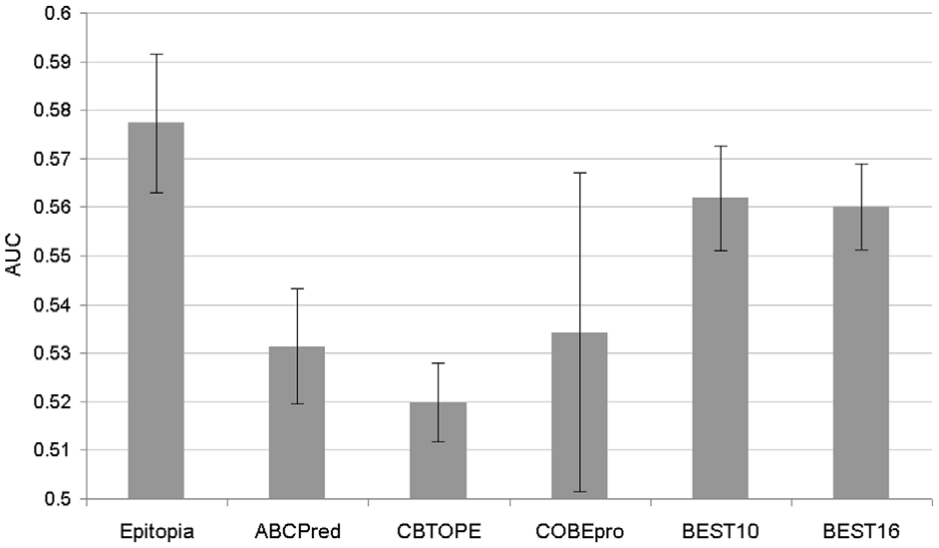
The average AUC values estimated using SEQ194 dataset. The values were calculated over the 10 repetitions using 100 randomly selected chains from the SEQ194 dataset (shown using gray bars) and the corresponding standard deviations (shown using black error bars) for the considered B-cell epitope predictors.

**Table 6:** Comparison of the proposed BEST method with existing B-cell epitope predictors on the SEQ149 dataset.

Category	Method	Success rate	AUC	Significance of improvement in AUC	
				compared to BEST_16_ g	compared to BEST_10_ g
Structure- based	Epitopia^a^	80.4%	0.59	unavailable	unavailable
	Epitopia^b^	73.7%	0.57	−	−
Sequence- based	ABCPred^a^	67.0%	0.55	unavailable	unavailable
	ABCPred^c^	61.9%	0.53	+	+
	BayesB^d^	80.9%	unavailable	unavailable	unavailable
	CBTOPE^e^	45.9%	0.52	+	+
	COBEpro^a^	66.9%	0.55	unavailable	unavailable
	COBEpro^f^	66.3%	0.54	+	+
	BEST _10_ g	58.8%	0.57		
	BEST _16_ g	60.3%	0.57		

The methods are sorted alphabetically within each category. We evaluate significance of differences between BEST_16_ (BEST_10_) and the other methods. We compare the corresponding AUC values in 10 paired results based on 100 random selected chains from the SEQ194 dataset using paired t-test; +/– mean that BEST_16_ (BEST_10_) are significantly better/worse that another method at *p*-value <0.05.

a results from [34].

b results from the Epitopia web server at http://epitopia.tau.ac.il/.

c results from the ABCPred web server http://www.imtech.res.in/raghava/abcpred/.

d results from the BayesB web server at http://www.immunopred.org/bayesb/index.html.

e results from the CBTOPE web server at http://www.imtech.res.in/raghava/cbtope/.

f results from the COBEpro web server at http://scratch.proteomics.ics.uci.edu/.

g results generated using BEST method, which is based on the SVM model (*C* = 1.0 and *gamma* = 0.001953) with 84 features generated with the Filtered40_BCPREDFrag dataset and the distance scheme with *k* = 16 (BEST_16_) and with *k* = 10 (BEST_10_).

We calculate the area under the ROC curve (AUC) to evaluate the real-valued predictions. We also use the success rate that was proposed earlier [34], [35]. The success rate is defined by the number of correctly predicted proteins divided by the total number of predicted proteins. A given chain is assumed to be correctly predicted if the average of the real-valued predicted propensities for the native epitope residues is larger than the average real-valued predicted propensities of all residues in that chain.

**Figure 6:**
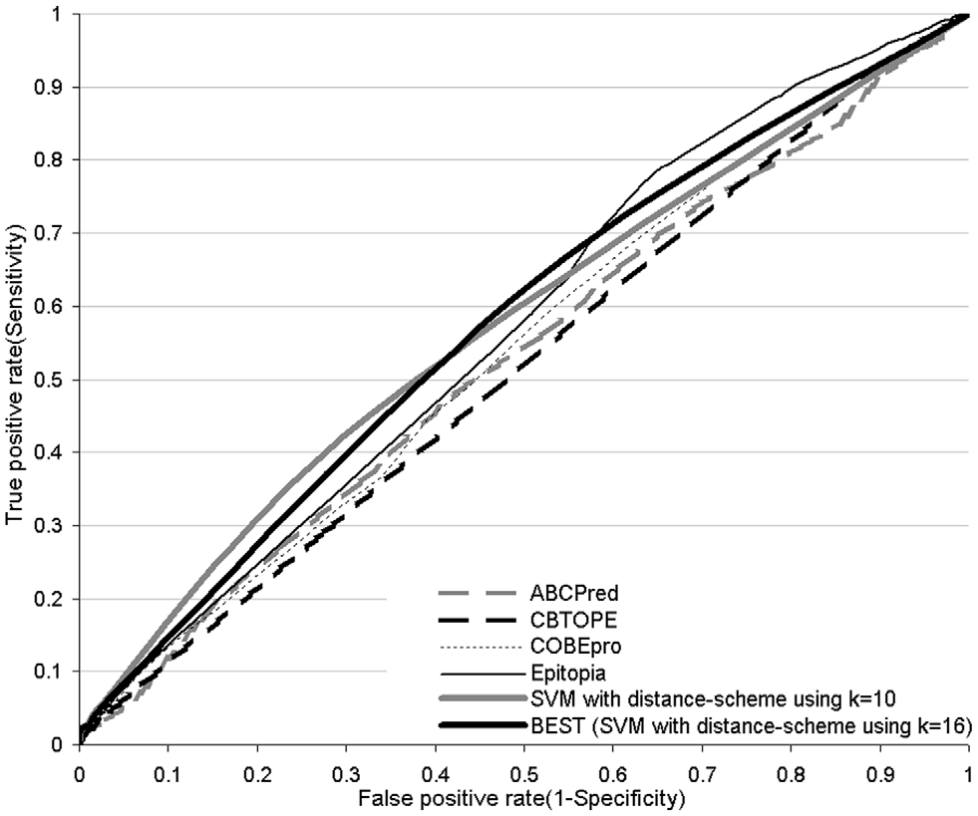
Receiver operating characteristic (ROC) curves of the considered B-cell epitope predictors on the SEQ194 dataset.

### Feature-based representation of the input sequence

We considered five types of input information to calculate our features: predicted secondary structure, predicted solvent accessibility, dipeptides-based antigenicity scale, and the conservation and similarity scores.

**Figure 7:**
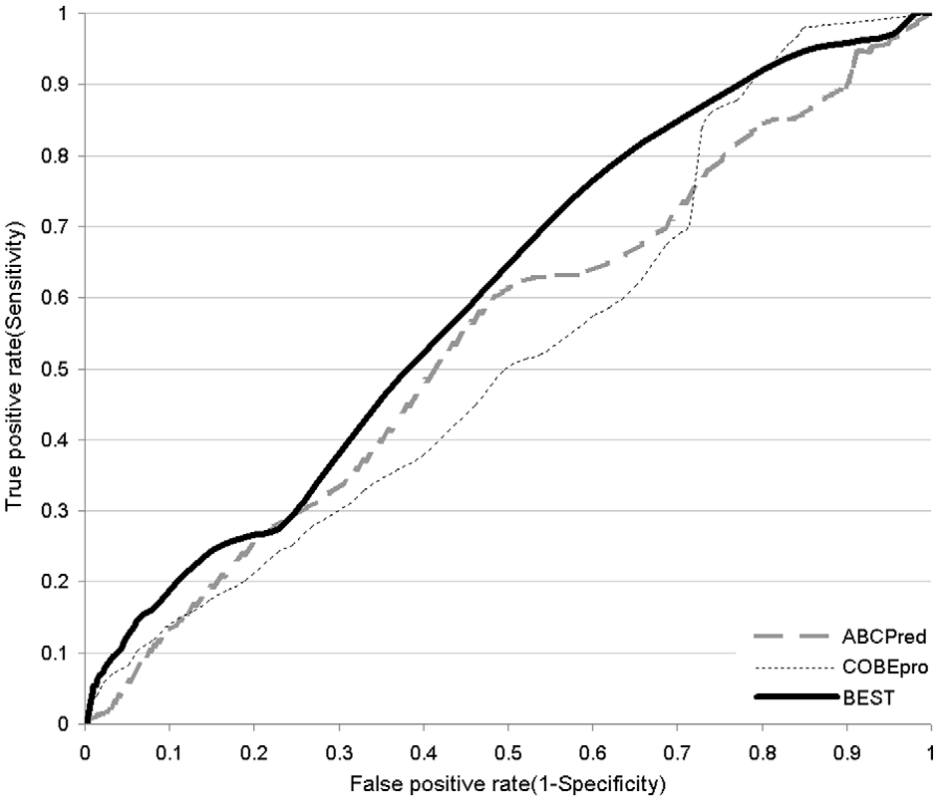
Receiver operating characteristic (ROC) curves of the considered B-cell epitope predictors on the SEQ19 dataset.

Secondary structure and solvent accessibility were predicted with the standalone version 3.0 of Real-SPINE [36]. We use relative solvent accessibility (RSA), which is defined as the ratio of solvent accessible surface area (ASA) of a residue observed in its three dimensional structure to that observed in an extended tripeptide conformation. We normalize the ASA values generated by Real-SPINE using Ala-X-Ala tripeptide as suggested in [41], [42]. The RSA values were used to categorize residues as buried (if predicted RSA<25%) or solvent exposed (otherwise).

**Figure 8:**
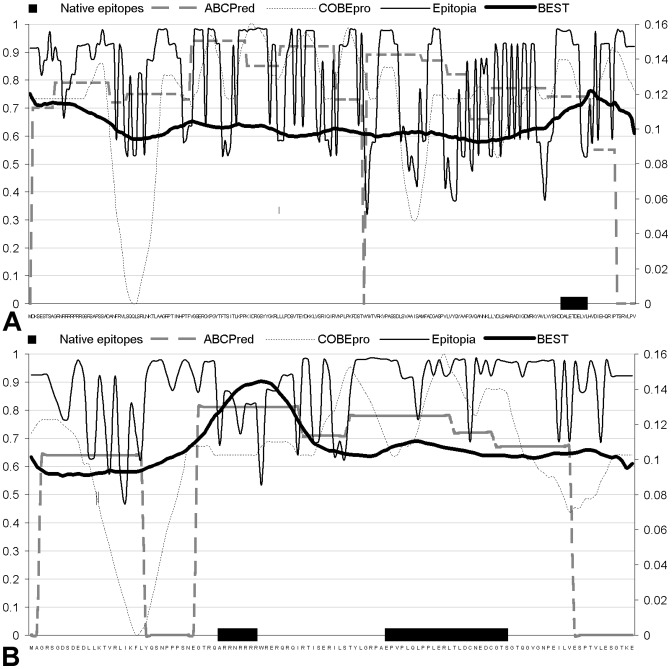
Residue epitopic propensities predicted by ABCPred, COBEpro, Epitopia and BEST for a capsid protein (UniProt ID: P16489; panel A) and an anti-repression transactivator protein (UniProt ID: P20869; panel B). The plots also include the location of the native epitopes. The *x*-axis shows the protein chain and the location of the native epitopes (denoted with black horizontal line) and *y*-axis shows the values of the predicted propensities. The left *y*-axis gives the propensities for ABCpred, COBEpro and Epitopis and the right *y*-axis for BEST.

The amino acid pair propensity scale (AAP) was first introduced by Chen et al. [20]. This scale quantifies propensity of a given dipeptide (AA pair) to form B-cell epitope and was shown to provide useful information to predict B-cell epitopes [20]. The original AAP values were renormalized to the (−1, 1) interval [21] and we denote them as the RAAP scale.

We run PSI-BLAST [24] against the nr dataset using default parameters (-j 3, -d nr) to compute the conservation which is defined as [43]:

Conservation  =  SUM*_i = 1..20_* { P_i_*log_2_(*P_i_/P_ib_*}

where *P_i_* is the value from the Weighted Observation Percentage (WOP) matrix generated by PSI-BLAST, which is divided by 100, and *P_ib_* is the background probability of each of the 20 AAs. If for a given residues all WOP values equal zero, i.e., *Pi* is a vector of 20 zeroes, then we use the average WOP values that are computed as the average over all residues of the same type in the training dataset for which the WOP values are non-zero. The selection of this conservation measure is motivated by results in [43].

Following [22], we compute similarity scores that quantify similarity of a given input 20-mer and the epitope and non-epitope fragments in the corresponding training dataset; we adjust the training datasets for each fold in the cross-validation tests and we use Filtered40_BCPREDFrag dataset when testing on the SEQ194 dataset. The scores are based on the total number of identical substrings (multi-mers) between the two 20-mers, i.e., they count the number of the same AAs, the same 2-mers, 3-mers, etc. present in both fragments. Such scores were found to be the most effective among several possible similarity measures in [22]. We use the five highest scores when calculating similarity to epitope fragments and non-epitope fragments, respectively.

Using these above information, we considered the following 11 groups of features:

1. *Secondary structure-based (8 features).*
  - *content_ss_* is the content (fraction) of the residues in the input 20-mer that have a given predicted secondary structure *ss*  =  {helix (H), strand (E), coil (C)} (3 features).
  - *entropy_SS*  =  SUM*_ss = {helix,strand,coil}_*{*content_ss_*ln(*content_ss_*)}, which is the overall entropy of the predicted secondary structure in the input 20-mer (1 feature).
  - *NumSeg_ss_* is the number of segments of a given predicted secondary structure type *ss* in the input 20-mer. A segment is defined as a stretch of consecutive AAs with the same secondary structure. For example, for the predicted secondary structure “HHHCEEEEEEEECCCHHHCCCECC”, *NumSeg_H_* = 2, *NumSeg_C_* = 4, *NumSeg_E_* = 2. (3 features).
  - *NumSeg_SS* is the total number of predicted secondary structure segments in the input 20-mer (1 feature).

We note that similar, segment-based features were successfully used in [44].

1. *RSA-based (33 features).*
  - *content_Bd/Ed_* is the content (fraction) of the residues in the input 20-mer that that are predicted to be buried (Bd) or solvent exposed (Ed) (2 features).
  - *entropy_RSA*  =  SUM*_i = {buried,exposed}_*{*content_i_*ln(*content_i_*)}, which is the overall entropy of the predicted solvent exposure (content of buried vs. solvent exposed residues) in the input 20-mer (1 feature).
  - *RSA_Bd/Ed_* is the average predicted RSA value for buried (Bd) or solvent exposed (Ed) residues in the input 20-mer (2 features).
  - *max/min_RSA_slide_n_* is the maximum/minimum value of predicted RSA averaged over a sliding window of size *n* = 5,6, …,17,18 within the input 20-mer. We consider 14 sizes of sliding window and calculate both min and max values (14×2 = 28 features). This allows us to find smaller fragments of input 20-mer that are either solvent exposed or buried.
2. *RAAP-based (30 features).*
  - *avg_RAAP* is the average RAAP value of the input 20-mer (1 feature).
  - *sd_RAAP* is the standard deviation of RAAP values of the input 20-mer (1 feature).
  - *max/min_RAAP_slide_n_* is the maximum/minimum value of RAAP averaged over a sliding window of size *n* = 5,6, …,17,18 within the input 20-mer (14×2 = 28 features).
3. *Conservation score-based (29 features.).*
  - *avg_CON* is the average conservation score of the input 20-mer (1 feature).
  - *max/min_CON_slide_n_* is the maximum/minimum value of conservation score averaged over a sliding window of size *n* = 5,6, …,17,18 within the input 20-mer (14×2 = 28 features).
4. *Secondary structure and RSA-based (12 features).*
  - *Num_ss_Bd/Ed_* is the number of residues in the input 20-mer that have a given predicted secondary structure *ss* and which are predicted to be buried (Bd) or solvent exposed (Ed) (3×2 = 6 features).
  - *RSA_ss_* is the average predicted RSA value for the residues in the input 20-mer that are predicted to have secondary structure *ss* (3 features).
  - *RSA_max_segment_ss_* is the average predicted RSA value for the longest segment of a given predicted secondary structure type *ss* in the input 20-mer (3 features).
5. *Secondary structure and conservation score-based (6 features).*
  - *CON_ss_* is the average conservation value for residues in the input 20-mer that have a given predicted secondary structure *ss* (3 features).
  - *CON_max_segment_ss_* is the average conservation value for the longest segment of a given predicted secondary structure type *ss* in the input 20-mer (3 features).
6. *Secondary structure and RAAP-based (6 features).*
  - *RAAP_ss_* is the average RAAP value for residues in the input 20-mer that have a given predicted secondary structure *ss* (3 features).
  - *RAAP_max_segment_ss_* is the average RAAP value for the longest segment of a given predicted secondary structure type *ss* in the input 20-mer (3 features).
7. *RAAP and RSA-based (30 features).*
  - *RAAP_Bd/Ed_* is the average RAAP value of the predicted buried (Bd) or solvent exposed (Ed) in the input 20-mer (2 features).
  - *avg_RAAP_max/min_RSA_sliden*, is the average RAAP value in a sliding window of size *n* = 5,6, …,17,18 within the input 20-mer that has the maximum/minimum average predicted RSA value (14×2 = 28 features).
8. *RAAP and conservation score-based (28 features).*
  - *avg_RAAP_max/min_CON_sliden* is the average RAAP value in a sliding window of size *n* = 5,6, …,17,18 within the input 20-mer that has the maximum/minimum average conservation score value (14×2 = 28 features).
9. *Secondary structure, RAAP and RSA-based (6 features).*
  - *RAAP_ss_Bd/Ed_* is the average RAAP value for residues in the input 20-mer that have a given predicted secondary structure *ss* and which are predicted to be buried (Bd) or solvent exposed (Ed) (6 features).
10. *Similarity score-based (10 features).*
  - *max_similarity_epitope_k_* is the *k* ^th^ highest similarity score between the input 20-mer and the epitope fragments from the training dataset; *k* = 1,2,3,4,5 (5 features).
  - *max_similarity_non-epitope_k_* is the *k* ^th^ highest similarity score between the input 20-mer and the non-epitope fragments from the training dataset; *k* = 1,2,3,4,5 (5 features).

Table 1 summarizes the considered 198 features, which are divided into the above mentioned 11 groups. While some of these features use the information that was previously considered to predict B-cell epitopes, including predicted secondary structure and RSA, RAAP and similarity scores, we also use conservation scores that were not used by the prior sequence-based predictors. Moreover, we propose a novel set of features that combine multiple types of information (such as predicted secondary structure and RSA; predicted secondary structure and conservation, etc.) and we use of sliding window to find fragments of the input 20-mer (such as fragments with low/high RAAP score, RSA value, etc.) that are relevant to the prediction of the B-cell epitopes.

### Feature selection and parameterization of the SVM model

The considered features may include features that are not relevant to the prediction of B-cell epitopes and which could be correlated/redundant with each other. We perform a wrapper-based (using the SVM model) feature selection, to accommodate for the above. We use the SVM model with the RBF kernel and we parameterized it using a grid search considering the complexity constant *C* and the *gamma* (spread of the RBF function) using all 198 features. Parameterization was done based on the 10-fold cross validation on the training BCPREDFrag dataset and we considered *C* = 2^−2^,2^−1^ …, 2^3^,2^4^ and *gamma* = 2^−11^,2^−10^…,2^−1^,2^0^. The selected parameters are *C* = 2^3^ and *gamma*  = 2^−10^, and we use these parameters through the entire feature selection process.

We first sort all features based on their average (over the ten training folds generated based on the 10 fold cross-validation on the training dataset) absolute biserial correlation coefficients (BCC). The BCC is defined as:

BCC = (*M_e_*-*M_ne_*)*sqrt(n_e_*n_ne_/*n*)/(*stdev*)

where *M_e_* and *M_ne_* are the mean values of the feature values for native epitopic and non-epitopic residues, respectively, *stdev* is the standard deviation of the feature, *n_e_* and *n_ne_* are the numbers of native epitopic and non-epitopic residues, respectively, and *n* is the total number of residues.

Next, we iteratively try to remove one feature at the time starting with the entire set of 198 sorted features and considering the least correlated features first. We calculate MCC for the 10-fold cross validation-based prediction of B-cell epitopes on the training (BCPREDFrag) dataset using the SVM classifier with a given set of features. We remove a given feature if this removal does not lower the MCC value. We repeat this until none of the features can be removed, i.e., removal of any feature leads to a decrease in the MCC. This type of feature selection was motivated by similar approaches used in related studies [45]–[47].

Consequently, 84 features were retained, see Table 1. A detailed list of the selected features is given in Table S1. Importantly, the selected features cover each of the considered 11 feature groups, which suggests that all considered groups contribute to the prediction of B-cell epitopes. The largest subset of the selected features concerns the RAAP scale, 60 out of the selected 84 features use the RAAP values. The arguably best feature, which has the highest absolute BCC of 0.47 (compared to the second-best feature with the absolute BCC = 0.4), is the *max_similarity_epitope_1_*. This feature quantifies to the highest similarity score against the database of training B-cell epitopes. This agrees with the results in [22], where the authors demonstrate use of these similarity scores leads to relatively accurate predictions of the epitopes. The selected features also include 65 that are based on using sliding windows inside the 20-mers. This shows that the use of the sliding windows, which is proposed in this work, is beneficial when compared to the use of the entire 20-mer. Moreover, 44 of the selected features use information coming from multiple types of inputs, which points to the importance of the novel aspects introduced in this work. Finally, 21 features utilize information coming from the conservation scores, which indicates that this input, which we also introduced here, provides a valuable contribution.

We again parameterize the SVM model using the same grid search with the selected features. The selected parameters are *C* = 2^0^ and *gamma*  = 2^−9^, and we used these parameters to implement our BEST method and to perform predictions on all considered datasets.

### Calculation of propensity scores

The real-value outputs generated by the SVM model, which are calculated for the overlapping 20-mers extracted from the input protein chain and which approximate the probability of a given 20-mer to be a B-cell epitope, are used to calculate propensity of each AA to form of a B-cell epitope. We assign the same SVM score to every AA in a given 20-mer, which means that every AA in the input chain has between 1 (for the residues at either terminus) and 20 (for residues 20 or more positions away from a terminus) SVM scores assigned to it; these scores come from the overlapping 20-mers. We consider four schemes to calculate the propensity from these scores:

- *max scheme* in which we use the maximal score as the propensity. This scheme assumes that a given AA is likely to be an epitope if it was predicted as such (has a high SVM score) in even one 20-mer that includes it.
- *average scheme* in which we use an average score. In this case, we implement a consensus-like decision where the propensity is based on all corresponding scores generated by the SVM.
- *median scheme* in which we use a median score. This is again a consensus-like prediction but in this case we use one of the SVM scores, instead of calculating a new average value.
- *distance scheme* where we calculate an average score but considering only a subset of the SVM scores. This is a novel approach in which we use only higher quality SVM scores. We note that the predictions associated with either low or high scores are usually more accurate compared with the predictions that have scores close to 0.5, which is the cutoff to separate the two outcomes; the 20-mers with scores <0.5 and >0.5 are assumed not to be epitopes and to be epitopes, correspondingly. This was shown for related SVM-based predictors [48], [49]. Therefore, we use only *k* = 1,2, …,20 scores that are the farthest from 0.5 to compute the average; for *k* = 20 this is equivalent to computing the average-scheme. We estimate the best value of *k* empirically; see Section “Selection of the method to calculate propensity scores”.

## Results

### Comparison on the fragment-based datasets

We evaluate the results generated by our SVM models, using both the model with all 198 features and the model with the selected 84 features, on two benchmark fragment-based datasets: BCPREDFrag and ChenFrag. These datasets include 20-mers of epitopes and non-epitopes, which were generated by truncation-and-extension. We compare our predictions with the results of recent predictors, including the method by Chen et al. [20], BCPred [21], and COBEpro [22]. Table 2 summarizes the results based on the 10-fold cross validation on the BCPREDFrag dataset, while Table 3 shows results on the ChenFrag dataset; we use 10-fold or 5-fold cross validation to mimic the tests from the original papers. Table 2 indicates that our SVM model with 198 features achieves an AUC of 0.81, accuracy of 74.5% and MCC of 0.53 on the BCPREDFrag dataset. The model with the selected 84 features achieves similar predictive quality, with AUC, accuracy, and MCC of 0.81, 74.0% and 0.55, respectively. The same level of similarity between these two approaches is observed on the ChenFrag data set. This demonstrates that the reduction of the feature set does not worsen the overall quality of the prediction. We note that the model with more input features gives a better sensitivity as a trade-off for reduced specificity, which means that it predicts more native epitope fragments but with a higher number of false positives.

Compared with the other considered predictors, our SVM models achieve the best predictions with an AUC of 0.81 and 0.85 and the highest MCC of 0.55 and 0.63 on the BCPREDFrag and ChenFrag datasets, respectively. The second-best predictor, COBEpro, obtains an AUC of 0.77 and 0.83 and MCC of 0.45 and 0.59 on the BCPREDFrag and ChenFrag datasets, respectively. Our models are characterized by high specificity (they rarely confuse non-epitopes for epitopes), and sensitivity which is similar to the sensitivity offered by existing methods. The sensitivity in the 0.5 to 0.6 range means that about 50 to 60% of native epitopes are correctly predicted. The high precision offered by our SVM model with 84 features means that virtually all of the predicted epitopes are in fact correct. This means that our SVM-based approach provides predictions that are conservative, i.e., it predicts a subset of native epitopes but with high quality. We observe that the results on the ChenFrag dataset are better than for the BCPREDFrag dataset. This is since the former dataset includes chains with higher similarity (with each other) when compared with the latter dataset.

### Improvements due to the inclusion of novel features

We analyze the impact of the novel aspects that were introduced in this study, including the new features and the fact that we effectively combine multiple features, including new and previously proposed features. We compare the results of our SVM-based model with 84 features with the results obtained when using the RAAP scale from Chen et al. [20] and the similarity measure introduced in [22]. To do that, we developed two SVM-based predictors that use the *avg_RAAP* feature (denoted as *RAAP model*) and the *max_similarity_epitope_1_* feature (*MaxSimilarity model*), respectively. These are the two best ranked features (see Table S1) that utilize the concepts introduced in these two works. These two models were parameterized on the training BCPREDFrag dataset in the same way as the SVM models proposed in this work. Consequently, these two models are the same as the proposed SVM model, except for the input features. The ROC curves of the three models on BCPREDFrag and ChenFrag datasets are shown in Figure 2.

We observe that our model provides higher sensitivity (TP-rate) for the entire range of FP-rates (FP-rate  = 1-specificity). The AUC values of the RAAP and MaxSimilarity models on the BCPREDFrag dataset are 0.73 and 0.72, respectively, compared to 0.81 achieved by our model with 84 features. Similarly, the two single feature-based models obtain AUC equal to 0.74 and 0.79 on the ChenFrag dataset, while we obtain 0.85 when using all 84 features. This is a relatively large increase by 100%*(0.81–0.73)/0.5 = 16% and by 100%*(0.85–0.79)/0.5 = 12% on the BCPREDFrag and ChenFrag datasets, respectively, given that AUC values range between 0.5 (for random predictions) and 1 (for perfect predictions). We attribute this increase to the use of novel features and the combination of the new and existing features that are implemented in our approach.

We also investigate contributions of individual feature groups, which are defined in Table 1. Table 4 shows the AUC values when only the selected features in each of the considered feature groups are utilized. Almost all the considered feature groups lead to an AUC above 0.5, which means that these models are better than random and that the corresponding features contribute to the final model that fuses all these features; the only exception are the conservation score-based features which on its own reach AUC of 0.5. Moreover, we observe that our approach to expand ideas from the prior works is beneficial. For instance, the use of the 7 selected similarity score-derived features leads to improvements when compared to using only the one *max_similarity_epitope_1_* feature, which is based on [22]; the corresponding AUC values are 0.76 vs. 0.72 on the BCPREDFrag dataset and 0.82 vs. 0.79 on the ChenFrag dataset. Also, the use of the combined set of 84 features results in higher AUCs compared to the best performing individual feature group. Specifically, the best performing similarity score-based group provides AUC values lower by 0.053 and 0.024 on the BCPREDFrag and ChenFrag dataset, respectively, when compared to our SVM that used 84 features.

We further analyze the similarity-based scores between the 20-mers from the BCPREDFrag dataset and the library of the epitope fragments, i.e., the *max_similarity_epitope_1_* feature. We plot the values of this feature (see Figure 3) separately for the native epitope (using black line) and non-epitope (gray line) fragments. The plots demonstrate, as expected, that native epitopes have overall substantially higher similarity with each other compared to the similarity between non-epitopes and epitopes. The mean and variance of the scores for the native epitopic fragments are 45.8 and 1455.7, respectively, while they are 16.4 and 13.9 for the non-epitopic fragments. However, about 300 native epitopic fragments have scores that are low (<20) and comparable to the scores for the non-epitopic fragments. These fragments cannot be correctly predicted using the similarity score alone. We note that there are only a few non-epitopic 20-mers that have high similarity to the epitopic fragments. This provides a potential explanation for the high specificity offered by our SVM model.

### Selection of the method to calculate propensity scores

We compare the predictive quality for the considered four methods (see section “Calculation of propensity scores”) that calculate the propensity of residues in a protein sequence to form of a B-cell epitope based on scores predicted by our SVM model with 84 features using the sliding window of 20-mers. In other words, we chunk the input protein using a sliding window of size 20, process each window using our SVM model and combine the scores generates by the SVM using each of the four methods (*maximum, average, median* and *distance scheme*) to predict a full protein chain. First, we parameterize the *distance scheme* to select the number of scores, *k*, that will be used, see Figure 4. We perform the calculations on the SEQ194 dataset (we use the Filter40_BCPREDFrag to generate the SVM model) and we use AUC and success rate as the evaluation criteria. The results indicate that the predictive quality is higher when we choose *k* between 10 and 16. Using smaller *k* would remove some of the useful scores and using higher *k* would include too many scores which may include some poor quality predictions. We compare the *distance scheme* with *k* = 10 and *k* = 16 with the other three approaches in Table 5. The use of the *median scheme* results in the highest success rate at 60.8% and the third-best AUC of 0.55. The application of the *distance scheme* with *k* = 16 leads to the highest AUC equal 0.57 and the second-best success rate of 60.3%. Consequently, we select this *distance scheme* to compute the propensities and to implement our BEST method. Our predictor can be downloaded from http://biomine.ece.ualberta.ca/BEST/.

### Comparison on the sequence-based datasets

We compare our BEST method, which uses the SVM model with 84 features generated with the Filtered40_BCPREDFrag dataset and the distance scheme with *k* = 16, with recent representative sequence-based predictors of B-cell epitopes, including ABCPred [19], COBEpro [22], BayesB [23], and CBTOPE [25]. We also include the results from the structure-based predictor Epitopia [34], [35] and the alternative version of our method that uses *k* = 10. Since some methods only predict epitopic fragments in a protein chain, we computed the propensities for each amino acid as follows:

- For Epitopia, we utilized the immunogenicity scores generated by the web server at http://epitopia.tau.ac.il/, and we normalize them into [0,1] interval.
- For ABCPred, we used the web server at http://www.imtech.res.in/raghava/abcpred/ with default parameters. The server returns predicted epitopic fragments with their scores. For a given residue, we used the maximal score from all fragments where this residue is included.
- For COBEpro, we used the web server at http://scratch.proteomics.ics.uci.edu/ and we followed the procedure from [22].
- For BayesB, we performed predictions based on the web server at http://www.immunopred.org/bayesb/index.html. This method was designed to predict linear B-cell epitopes and it returns a list of predicted epitopes as 20-mers, with no scores. We assumed that a given residue is a B-cell epitope if it appears in at least one of the predicted 20-mers; otherwise, it is assumed not be an epitope. We could not calculate AUC for BayesB since this method does not return scores.
- For CBTOPE, we calculated the predictions with the web server at http://www.imtech.res.in/raghava/cbtope/ using default parameters. We divided the scores generated by the server, which are in 0 to 9 range, by 10 to normalize them into [0, 1] interval.

The comparison is performed on the SEQ194 dataset, see Table 6. For Epitopia, ABCPred and COBEpro we show the predictions that were generated with the author-provided web servers together with the results on the same dataset from [34]. We also evaluate significance of differences between our predictor and the other methods using their web server predictions. We select 100 chains at random from the SEQ194 dataset and repeat the evaluation 10 times using these subsets of sequences. We use paired-t-test to compare the resulting AUC values and the differences are assumed significant if *p*-value <0.05. The corresponding average AUCs and their standard deviations are shown in Figure 5.

When compared with the sequence-based methods using Table 6, BEST (which uses *k* = 16) achieves the best AUC  = 0.57. The second-best ABCPred and COBEpro methods achieve AUC around 0.55. The improvements in AUC offered by BEST have moderate magnitude but these differences are significant when compared with all chain-based methods including ABCPred, CBTOPE, and COBEpro. The structure-based Epitopia outperforms our sequence-based approach and obtains AUC of about 0.57 (or 0.59 in the original paper). The corresponding ROC curves are shown in Figure 6. We note that BEST offers highest TP-rates (sensitivity) for higher FP-rates, while our SVM-based design with distance scheme with *k* = 10 offers highest sensitivity for low FP-rates. Structure based Epitopia is the only method that outperforms our SVM-based approaches for FP-rates above 0.6. However, BEST is outperformed by COBEpro, BayesB, ABCPred, and Epitopia when considering the success rates. We note that BayesB obtains high success rate at 80.9%. However, this is a byproduct the fact that this method substantially overpredicts epitopes; 97.6% residues are predicted as epitopes by the BayesB method. We also compare with a “random” predictor, which uses a randomly generated score between 0 and 1 for each 20-mer fragment and which calculates the propensity scores using the distance scheme with *k* = 16. When evaluated with AUC, the random method is significantly worse than our BEST (*p*-value  = 5.5*10^−8^).

We also perform a second test on the SEQ19 dataset. This dataset is arguably too small to assess statistical significance, but it allows gauging the overall predictive quality. Our BEST method achieves AUC of 0.601, while ABCPred and COBEpro, which are the top two sequence-based runner-up methods on the SEQ149 dataset, obtain AUC of 0.541 and 0.525, respectively. The corresponding ROC curves are given in Figure 7 and they show that BEST provides higher sensitivity (TP-rate) for the FP-rates below 0.8 when compared to the other two sequence-based predictors.

### Case studies

We present two case studies to visualize the propensity profiles generated by various considered B-cell epitope predictors. We selected two proteins from the SEQ194 dataset, a capsid protein (UniProt ID: P16489) with one short continuous epitope, and anti-repression transactivator protein (UniProt ID: P20869) that has a discontinuous B-cell epitope composed of two segments. Figure 8 shows the propensities predicted by ABCPred, COBEpro, Epitopia and BEST together with the location of the native epitopes. The propensity profiles generated by BEST are smooth dues to the use of averaging of the SVM scores and the peaks denote predicted epitopes. BEST gives a peak around the location of the native epitope for the capsid protein and another peak in the vicinity of the N-terminus in that chain; the latter is a likely false positive prediction; see Figure 8A. For the anti-repression transactivator protein (see Figure 8B) our method correctly predicts the shorter of the two epitope segments and provides slightly elevated propensities for the longer segment. ABCpred managed to quite well identify the epitopes in the latter protein, but it could not find the epitope in the capsid protein. COBEpro and Epitopia find the longer epitope fragment in the anti-repression transactivator and several (potentially) false positive epitopes in both proteins. We note that these results should not be assumed to be typical, i.e., to represent “average” predictive quality across these methods which is summarized in Table 6; they are presented to contrast the overall characteristics of the propensity profiles generated by these methods.

## Discussion

We propose a new approach for the prediction of B-cell epitopes from antigen sequences. Our BEST method predicts epitopes from full protein chains using a novel approach based on averaging selected scores generated from 20-mers by an SVM-based predictor. We use a comprehensive and custom designed set of inputs that are generated by fusing information derived from the protein chain, similarity to known (training) epitopes, sequence conservation and predicted secondary structure and relative solvent accessibility. Empirical evaluation on benchmark datasets (including an independent test set of 194 antigens) demonstrates that BEST outperforms several modern sequence-based B-cell epitope predictors including ABCPred [19], method by Chen et al. [20], BCPred [21], COBEpro [22], BayesB [23], and CBTOPE [25], when considering the predictions from full chains and also from the chain fragments. We show that the improvements came from the design and use of new inputs, which include conservation scores. These scores and other inputs were combined together to calculate fused features. These individual features combine information from multiple inputs, e.g., one feature fuses information from the predicted secondary structure, sequence and sequence conservation. We also present a couple of case studies to demonstrate the propensity profiles generated by BEST.

The predictive quality offered by our method can be potentially further improved. One possibility is to first use the antigen sequence to predict its fold, which would be than used as an input. This is motivated by superior predictive performance of the structure-based predictors when compared to the sequence-based methods [3], [31], [34]. The structure could be also approximated with the use of sequence-predicted structural characteristics, such as contact numbers or B-factors [50], which are utilized by some of the structure-based predictors [27], [30], [32]. Another worthwhile input is disorder, and in particular molecular recognition features that are important for protein recognition [51] and which can be predicted from the sequence [52], [53]. However, the main limiting factor is the fact that only a small fraction (several thousand) of the epitopes is known and can be used to build predictive models compared to about a trillion antibodies in our body, when excluding T cell receptors [3]. We believe that major improvements can be accomplished only when additional data becomes available.

BEST can be downloaded from http://biomine.ece.ualberta.ca/BEST/.

## Supporting Information

Table S1 **List of the 84 selected features.** The features are sorted according to the average (over the ten training folds generated based on the 10 fold cross-validation on the training dataset) absolute biserial correlation coefficient (BCC).
(PDF)
