# Supplementary material for: BEST: Improved Prediction of B-Cell Epitopes from Antigen Sequences
Source: PLoS One. 2012 Jun 27;7(6):e40104. doi: 10.1371/journal.pone.0040104 (PMC3384636; doi:10.1371/journal.pone.0040104)
Supplement: Table S1 — List of the 84 selected features. The features are sorted according to the average (over the ten training folds generated based on the 10 fold cross-validation on the training dataset) absolute biserial correlation coefficient (BCC). (PDF) [file pone.0040104.s001.pdf]

Table S1. List of the 84 selected features. The features are sorted according to the average (over the ten training folds generated based on the 10 fold cross-validation on the training dataset) absolute biserial correlation coefficient (BCC).

Feature Name	BCC
$NumSeg_E$	0.0093
Num_{C_Bd}	0.0095
Num_{E_Ed}	0.0214
Num_{E_Bd}	0.0252
$min\_CON\_slide_5$	0.0269
$max\_CON\_slide_7$	0.0336
Num_{H_Ed}	0.0772
$max\_similarity\_non\text{-}epitope_3$	0.1273
$max\_similarity\_epitope_5$	0.1318
$NumSeg_H$	0.1442
$max\_similarity\_non\text{-}epitope_4$	0.1470
CON_H	0.1515
$max\_similarity\_non\text{-}epitope_5$	0.1581
$max\_similarity\_epitope_4$	0.1800
Num_{H_Bd}	0.1829
$min\_RSA\_slide_{11}$	0.1928
$max\_RSA\_slide_{17}$	0.1937
$min\_RSA\_slide_9$	0.1938
$max\_RSA\_slide_{12}$	0.1938
$min\_RSA\_slide_{17}$	0.1940
$RAAP_H$	0.2198
$RAAP_{C\_Ed}$	0.2269
$avg\_RAAP\_min\_CON\_\_slide_5$	0.2406
$avg\_RAAP\_min\_RSA\_slide_5$	0.2553
$avg\_RAAP\_max\_CON\_slide_6$	0.2689
$avg\_RAAP\_max\_RSA\_slide_8$	0.2766
$RAAP_{Ed}$	0.2850
$max\_similarity\_epitope_2$	0.3238
$avg\_RAAP\_min\_CON\_slide_{10}$	0.3335
$min\_RAAP\_slide_6$	0.3335
$avg\_RAAP\_max\_RSA\_slide_{11}$	0.3372
$max\_RAAP\_slide_9$	0.3388
$avg\_RAAP\_max\_CON\_slide_{11}$	0.3440
$min\_RAAP\_slide_7$	0.3452
$avg\_RAAP\_max\_CON\_slide_{12}$	0.3471
$avg\_RAAP\_min\_RSA\_slide_{12}$	0.3519
$avg\_RAAP\_max\_RSA\_slide_{13}$	0.3537
$max\_RAAP\_slide_{10}$	0.3538
$avg\_RAAP\_min\_RSA\_slide_{13}$	0.3558
$min\_RAAP\_slide_8$	0.3579

$avg\_RAAP\_max\_RSA\_slide_{14}$	0.3602
$min\_RAAP\_slide_{9}$	0.3616
$avg\_RAAP\_max\_RSA\_slide_{12}$	0.3629
$max\_RAAP\_slide_{11}$	0.3633
$avg\_RAAP\_min\_CON\_slide_{12}$	0.3651
$avg\_RAAP\_min\_RSA\_slide_{15}$	0.3657
$avg\_RAAP\_min\_RSA\_slide_{14}$	0.3664
$avg\_RAAP\_min\_CON\_slide_{16}$	0.3672
$avg\_RAAP\_min\_CON\_slide_{14}$	0.3676
$avg\_RAAP\_max\_CON\_slide_{13}$	0.3680
$min\_RAAP\_slide_{10}$	0.3707
$avg\_RAAP\_min\_CON\_slide_{17}$	0.3718
$avg\_RAAP\_min\_CON\_slide_{15}$	0.3724
$max\_RAAP\_slide_{12}$	0.3735
$avg\_RAAP\_max\_RSA\_slide_{16}$	0.3738
$avg\_RAAP\_max\_RSA\_slide_{17}$	0.3740
$avg\_RAAP\_max\_CON\_slide_{14}$	0.3748
$min\_RAAP\_slide_{13}$	0.3775
$min\_RAAP\_slide_{14}$	0.3778
$avg\_RAAP\_min\_CON\_slide_{13}$	0.3786
$max\_RAAP\_slide_{13}$	0.3789
$avg\_RAAP\_max\_RSA\_slide_{15}$	0.3790
$min\_RAAP\_slide_{12}$	0.3798
$max\_RAAP\_slide_{14}$	0.3802
$avg\_RAAP\_max\_CON\_slide_{15}$	0.3806
$min\_RAAP\_slide_{11}$	0.3808
$avg\_RAAP\_min\_CON\_slide_{18}$	0.3820
$avg\_RAAP\_min\_RSA\_slide_{16}$	0.3829
$avg\_RAAP\_max\_CON\_slide_{17}$	0.3832
$avg\_RAAP\_max\_RSA\_slide_{18}$	0.3832
$max\_RAAP\_slide_{15}$	0.3833
$max\_RAAP\_slide_{16}$	0.3837
$min\_RAAP\_slide_{16}$	0.3840
$avg\_RAAP\_min\_RSA\_slide_{17}$	0.3842
$min\_RAAP\_slide_{15}$	0.3848
$max\_RAAP\_slide_{17}$	0.3861
$max\_RAAP\_slide_{18}$	0.3867
$avg\_RAAP\_max\_CON\_slide_{16}$	0.3877
$min\_RAAP\_slide_{17}$	0.3881
$avg\_RAAP\_max\_CON\_slide_{18}$	0.3882
$avg\_RAAP\_min\_RSA\_slide_{18}$	0.3889
$min\_RAAP\_slide_{18}$	0.3890
avg_RAAP	0.3999
$max\_similarity\_epitope_{1}$	0.4686
